# Alcohol’s Actions on Neuronal Nicotinic Acetylcholine Receptors

**Published:** 2006

**Authors:** Tiffany J. Davis, Christopher M. de Fiebre

**Affiliations:** Tiffany J. Davis is a graduate student and Christopher M. de Fiebre, Ph.D., is an associate professor in the Department of Pharmacology and Neuroscience, University of North Texas Health Science Center, Fort Worth, Texas

**Keywords:** Alcohol and tobacco, alcoholism, cigarette smoking, nicotine dependence, brain, neuronal nicotinic acetylcholine receptors, cross-tolerance, alcohol and other drug (AOD) sensitivity, genetic factors, genetic polymorphism, laboratory mice, laboratory rat, neurotoxicity

## Abstract

Although it has been known for many years that alcoholism and tobacco addiction often co-occur, relatively little information is available on the biological factors that regulate the co-use and abuse of nicotine and alcohol. In the brain, nicotine acts at several different types of receptors collectively known as nicotinic acetylcholine receptors (nAChRs). Alcohol also acts on at least some of these receptors, enhancing the function of some nAChR subtypes and inhibiting the activity of others. Chronic alcohol and nicotine administration also lead to changes in the numbers of nAChRs. Natural variations (i.e., polymorphisms) in the genes encoding different nAChR subunits may be associated with individual differences in the sensitivity to some of alcohol’s and nicotine’s effects. Finally, at least one subtype of nAChR may help protect cells against alcohol-induced neurotoxicity.

It has been known for a long time that use and abuse of alcohol and tobacco products commonly occur together, as evidenced by the Reverend George Trask’s 1860 *Letters on Tobacco, for American Lads*, in which he wrote “Do you know of one drunkard that does not use tobacco?” (Trask 1860, p. 28). Today, smoking is recognized as one of the greatest risk factors in the development of alcoholism. Nevertheless, relatively little is known about the biological mechanisms underlying the co-abuse of tobacco products and alcohol. Nicotine is the principal addictive component of tobacco smoke, and researchers have identified specific protein molecules (i.e., receptors) in the brain at which nicotine acts. These receptors are collectively known as neuronal nicotinic acetylcholine receptors (nAChRs) because they primarily interact with the brain chemical (i.e., neurotransmitter) acetylcholine.

Even though alcohol has been shown to act directly on specific subtypes of these nAChRs, few studies have investigated the role of these receptors in modulating alcohol’s effects on the brain, and this issue remains an understudied and underfunded area of alcohol research. This article reviews the current state of knowledge regarding nAChRs and the interactions between alcohol and nicotine at these receptors, focusing on those nAChR subtypes that appear to be involved in modulating the actions of alcohol.

## nAChRs—Receptors for Nicotine

Nicotine interacts with several different nAChR subtypes in the brain. All of these nAChRs belong to a family of receptors that are collectively called ligand-gated ion channel receptors. Ions are atoms or molecules that carry an electrical charge because they possess different numbers of negatively (electrons) and positively (protons) charged particles. Common examples of ions include sodium (Na^+^),^1^ chloride (Cl^−^), potassium (K^+^), and calcium (Ca^2+^). The concentration of ions on the inside of neurons (nerve cells) versus the outside of neurons is strictly regulated. Typically, the concentration of K^+^ ions is greater inside the neuron than on the outside, whereas the concentrations of Na^+^, Cl^−^, and Ca^2+^ are greater outside the neuron than on the inside. Ligand-gated ion channel receptors, such as nAChRs, form pores or channels that, when opened, allow specific ions to flow into or out of neurons. For example, acetylcholine and certain drugs, such as nicotine, cause nAChRs to open (i.e., they “gate” the channel), thereby allowing Na^+^ and/or Ca^2+^ to enter a neuron. The resulting transient change in the internal ion concentration modulates the neuron’s excitability—that is, its ability to fire and transmit a nerve signal by releasing neurotransmitters that act on other neurons. In addition, the changes in ion concentrations provide specific signals or information to the affected neuron.

Neuronal nAChRs are made up of five proteins, or subunits, each of which traverses the cell membrane. Together, the five subunits form a complex around a central pore or channel, similar to staves around a barrel (see Figure). When the channel is opened, ions can flow into or out of the cell.

Several types of nAChR subunits exist. In the case of nAChRs in the brain, the subunits can be classified into two families, alpha (α) and beta (β). Each of these families has several members that are labeled in a numerical fashion (i.e., α2, α3, α4 ...α10 and β2, β3, or β4).^2^ Different combinations of these subunits result in the formation of different subtypes of nAChRs. Each nAChR subtype is named according to the subunits of which it is made up. For example, receptors of the α7 nAChR subtype each consist of five α7 subunits (see Figure, *top*). Receptors such as this, which comprise only a single type of subunit, are called homo-oligomeric receptors. Other nAChR subtypes are made up of two or more different subunits; these are known as heteromeric receptors. For example, each receptor of the α4β2 nAChR subtype consists of two α4 subunits and three β2 subunits (see Figure, *bottom*).

The composition of the various nAChR subtypes determines which ions can pass through the channels once they are opened. Thus, the α7 nAChR subtype principally allows Ca^2+^ to flow into the cell when the channel opens. Conversely, the α4β2 nAChR subtype allows both Na^+^ and Ca^2+^ to flow into the cell.

Of the numerous nAChR subtypes that exist, the α7 and α4β2 nAChR subtypes are the two most prevalent ones in the brain. Of these, the α4β2 nAChR subtype has a higher affinity for nicotine. This means that nicotine binds to this receptor at a lower concentration than is required for the α7 nAChRs.

**Figure:**
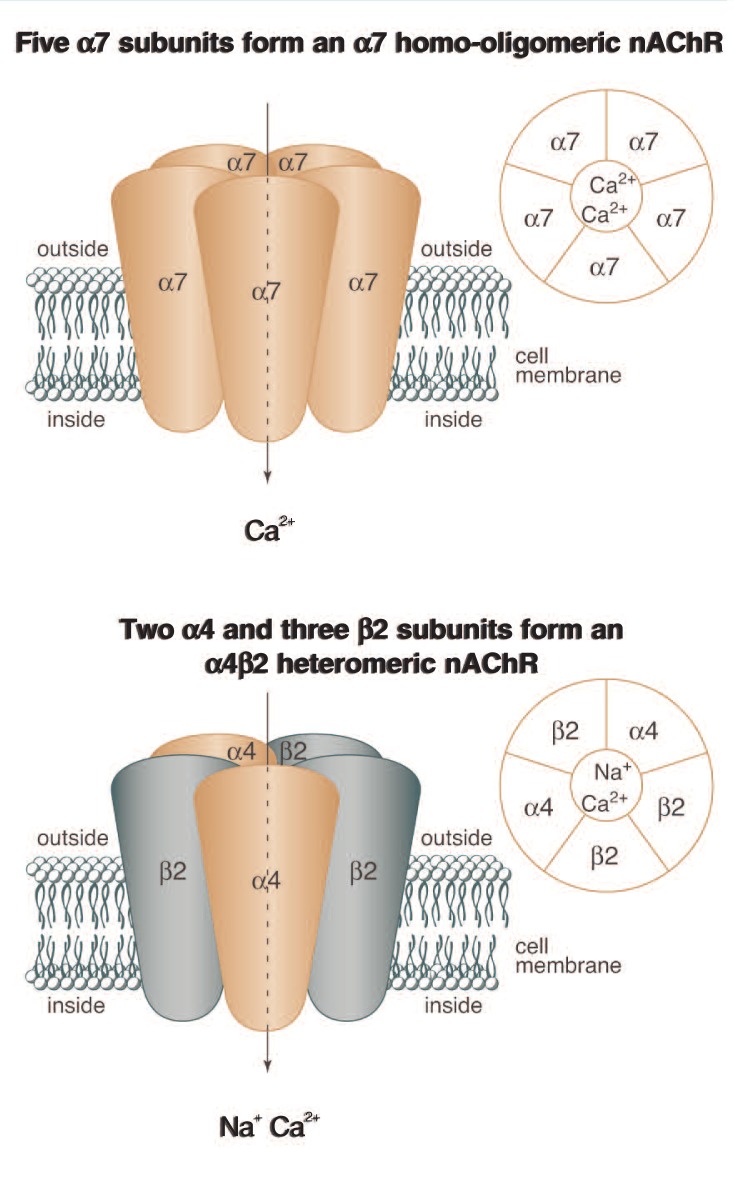
Schematic representation of the two most common subtypes of nAChRs. Both receptors consist of five subunits. The α7 nicotinic acetylcholine receptor (nAChR) consists of five α7 subunits and is called an α7 homo-oligomeric nAChR (*top*). The α4β2 nAChR is composed of two α4 subunits and three β2 subunits and thus is called an α4β2 heteromeric nAChR (*bottom*). In both nAChRs, the subunits are arranged around a central pore or channel that opens when agents such as acetylcholine or nicotine bind to the nAChR, allowing positively charged ions to flow through the channel into the cell. The α7 nAChR principally allows passage of calcium (Ca^2+^) ions, whereas the α4β2 nAChR allows passage of both calcium and sodium (Na^+^).

Most work in the alcohol–nicotine field has focused on the 4β2 and α7 nAChR subtypes, and this review will mostly focus on these. However, there also has been much interest recently in nAChR subtypes that interact with and are blocked by a substance called α-conotoxin MII (α-CtxMII)—a toxin isolated from certain sea snails. As described in the Sidebar, these receptors may mediate not only some of nicotine’s effects but may also modulate some of the rewarding properties of alcohol (Larsson et al. 2004).

## Alcohol’s Actions on Specific nAChR Subtypes

Alcohol and nicotine both act on the brain, and because so many people use and abuse both drugs it is likely that both drugs act on at least some of the same brain structures. One of the most probable places for alcohol and nicotine to interact is at nAChRs. Over the last two decades, numerous studies have shown that alcohol affects many different types of ion channels, including ligand-gated ion channel receptors (Narahashi et al. 2001). Moreover, researchers have demonstrated that alcohol can directly act on different nAChR subtypes. Many of these studies have been done using cloned receptors that were introduced into and produced (expressed) in frog eggs (*Xenopus* oocytes). Because these eggs do not normally express nAChRs, they provide a model system in which only the introduced nAChR subtype is produced. This design allows investigators to study, for example, alcohol’s or nicotine’s effects on one specific nAChR subtype without having to distinguish between diverse effects on different receptor subtypes.

Cardoso and colleagues (1999) have used such an oocyte system to express different human nAChR subtypes and study alcohol’s effects on them. The investigators found that alcohol enhances the function of several nAChR subtypes (i.e., α4β2, α4β4, α2β2, and α2β4) but has little effect on the function of other subtypes (i.e., α3β2 and α3β4). Moreover, studies in which either human or rat α7 nAChRs were expressed in oocytes demonstrated that alcohol inhibits this receptor subtype (Yu et al. 1996; de Fiebre and de Fiebre 2005; Cardoso et al. 1999).

Although frog oocytes offer a convenient way to study individual nAChRs, they are an artificial experimental system and one cannot exclude the possibility that the artificially expressed receptors differ in structure or function from those naturally expressed in brain cells. Therefore, Narahashi and colleagues (1999, 2001) also have studied nAChRs in neurons that were isolated from rat cerebral cortex and then grown in a cell culture dish. These studies demonstrated that naturally expressed nAChRs also are affected by alcohol. By treating neuronal cultures with several different drugs that have known effects on different nAChR subtypes, the researchers were able to determine which nAChR subtypes were affected by alcohol. Based on these analyses, the investigators concluded that both α4β2 nAChRs and α7 nAChRs were affected by alcohol. These studies confirm the results obtained using frog eggs, demonstrating that alcohol enhanced the function of naturally expressed α4β2 nAChRs and inhibited the activity of naturally expressed α7 nAChRs.

## Interactions of Alcohol and Nicotine at nAChRs

Nicotine’s effects at nAChRs are complex. Nicotine not only activates nAChRs but also can quickly inactivate these receptors via a process called desensitization.^3^ In fact, Brody and colleagues (2006) recently reported that with the amount of nicotine consumed by most cigarette smokers, the majority of α4β2 nAChRs should be in a continuous state of desensitization. It is not clear whether the nicotine-induced desensitization of nAChRs causes a smoker to no longer experience some of the effects of nicotine or if it actually produces an effect that smokers seek. Interestingly, Marszalec and colleagues (1999) have shown that alcohol interferes with the nicotine-induced desensitization of α4β2 nAChRs. As a result, alcohol may reverse some of the desensitization caused by smoking at these nAChRs. Whether this contributes to the co-use of alcohol and nicotine is not known.

By enhancing or inhibiting the function of different nAChR subtypes, alcohol not only affects normal signal transmission at these receptors by the neurotransmitter acetylcholine but also affects nicotine-induced signaling processes. It is plausible that such interactions between alcohol and nicotine at nAChRs may contribute to the common co-use of alcohol and tobacco products. However, researchers have not yet conclusively demonstrated if and how these interactions contribute to this most common form of polydrug abuse. What has been shown, however, is that chronic use of both alcohol and nicotine can alter the number of nAChRs in the brain.

## Modulation of nAChR Numbers by Alcohol and Nicotine

Studies conducted in the early 1980s first demonstrated that chronic nicotine treatment can cause an increase in the number of nAChRs in the brains of rodents. Moreover, several of these studies indicated that genetic factors influence the degree to which nicotine increases nAChR numbers in a given individual. Finally, the degree of increase differed among different brain regions and nAChR subtypes (Marks et al. 1991). In general, nicotine induced greater increases in α4β2 nAChR numbers than in α7 nAChR numbers.

Similar findings have been obtained in studies using human brain tissue obtained postmortem. For example, Teaktong and colleagues (2004) demonstrated that the number of α4- and α7-containing nAChRs differed between smokers and nonsmokers. As was expected based on earlier studies using rodent and human tissue, the effects of smoking on α4- and α7-containing nAChRs were independent of each other and differed among the brain regions examined. The most prevalent finding was that the number of nAChRs (particularly α4-containing nAChRs) was increased in smokers compared with nonsmokers; however, nAChR levels were decreased in some brain regions in smokers. In addition, the investigators discovered that even within a single neuron nicotine’s effects on nAChRs could vary depending on the specific cell region where the receptors were located.

Fewer studies have examined alcohol’s effects on nAChR numbers. Booker and Collins (1997) conducted a study using the long-sleep (LS) and short-sleep (SS) mice that were selectively bred to show particularly high or low sensitivity to some of alcohol’s effects. Thus, LS mice are more sensitive to alcohol’s sedative effects (i.e., they “sleep” longer after receiving a single high dose of alcohol) than are SS mice. In the study by Booker and Collins (1997), LS and SS mice were given alcohol in their drinking water for 6 months. This treatment caused changes in α4β2 and/or α7 nAChR numbers in only a few brain regions; most brain regions showed no change in nAChR numbers following chronic alcohol treatment. The changes that were observed, however, differed for LS and SS mice. This finding demonstrates that genetic factors influence the ability of chronic alcohol to affect nAChR numbers.

More recently, Dohrman and Reiter (2003) studied alcohol’s effects on nAChR numbers using cultured cell lines that express different nAChR subtypes. These researchers found that alcohol not only affects nAChR numbers by itself but also can modulate the degree to which nicotine can change nAChR numbers.

A few studies have tried to determine whether the alcohol- and nicotine-induced changes in nAChR numbers are responsible for the development of tolerance (i.e., decreased sensitivity) to alcohol and nicotine. Chronic alcohol treatment can produce not only tolerance to the effects of alcohol but also cross-tolerance to the effects of nicotine. Similarly, chronic treatment with nicotine can produce both tolerance to the effects of nicotine and cross-tolerance to the effects of alcohol (e.g., de Fiebre et al. 1990). The development of both tolerance to either drug and cross-tolerance between the two drugs is modulated by genetic factors, and, as described above, alcohol-induced changes in nAChR numbers depend at least in part on genetic factors. The evidence gathered to date, however, does not support the hypothesis that large changes in nAChR numbers are responsible for the development of tolerance to alcohol or cross-tolerance between alcohol and nicotine. Nevertheless, it is possible that more subtle changes in nAChRs numbers or composition may play a role in these processes. More research is needed to determine whether and how chronic treatment with alcohol and nicotine, either alone or together, modifies nAChR numbers, the expression of different nAChR subtypes, and the function of those nAChRs that are expressed following chronic treatment.

## The Role of Genes for nAChRs in Determining Alcohol and Nicotine Effects

For many years, it has been established that a person’s risk of becoming alcohol dependent is determined in part by that person’s genetic makeup. More recently, researchers also demonstrated that the risk of becoming a smoker (nicotine dependent) is determined in part by a person’s genetic makeup as well. The common co-occurrence of drinking and smoking leads to the question of whether the same or similar genes control the development of both alcohol and nicotine dependence.

To assess whether the genes that control sensitivity to certain effects of alcohol also control some aspect of sensitivity to the effects of nicotine, researchers have conducted studies with mice and rats selectively bred to differ in sensitivity to a specific action of alcohol, such as the LS and SS mice described above and their rat equivalents, the high alcohol sensitivity (HAS) and low alcohol sensitivity (LAS) rats (de Fiebre and Collins 1992; de Fiebre et al. 2002).

These studies found that animals with high sensitivity to alcohol’s sedative effects (i.e., LS mice and HAS rats) also were more sensitive to some of nicotine’s effects than animals with low sensitivity to alcohol’s effects (i.e., SS mice and LAS rats). These results suggest that there is some, albeit not complete, overlap in the genes that control sensitivity to both alcohol and nicotine. Numbers of nAChRs do not appear to control the differential sensitivity of LS versus SS mice or HAS versus LAS rats to either acute alcohol or nicotine. It is possible, however, that differences in the genes encoding the nAChR subunits may be responsible for differences in sensitivity to alcohol and nicotine.

### Naturally Occurring Alternate Forms of nAChR Subunit Genes

The statement that individuals differ in their genetic makeup does not mean that they carry completely different genes. For example, it would not be expected that one individual would have a gene for the β2 nAChR subunit and another person would have a gene for the β4 nAChR subunit in its place. Instead, both individuals would carry the genes for both the β2 and β4 nAChR subunits, but the chemical makeup of the β*2* and β*4 nAChR* genes may differ slightly between the two individuals. These variations within a gene, which are known as polymorphisms, could result in differences in the protein structure of the β2 and β4 nAChR subunit. These structural differences, in turn, could lead to differences in how the β2- or β4-containing nAChRs respond to nicotine, alcohol, or other drugs and, consequently, in a person’s sensitivity to these drugs. Researchers have investigated the effects of naturally occurring polymorphisms in both α4 and α7 nAChR subunits.

#### Polymorphisms in α4 nAChR Subunits

Stitzel, Collins, and colleagues have studied two polymorphic forms of the α4 nAChR subunit (Dobelis et al. 2002). The genes encoding these two alternate forms differ at a single site, resulting in a difference in one of the 628 amino acids that make up each α4 nAChR subunit protein. Despite this apparently minor change in the subunit protein, nAChRs containing these different forms of the α4 subunit differ both in how well nicotine activates these receptors (Dobelis et al. 2002) and in the ability of alcohol to enhance the function of these receptors (Butt et al. 2003). Interestingly, these alternate forms of the α*4 nAChR* subunit gene were first identified in the LS and SS mice (Stitzel et al. 2001). Whether the α*4 nAChR* polymorphisms are responsible, at least in part, for the differences between LS and SS mice in sensitivity to both alcohol and nicotine remains unknown. Studies in so-called recombinant inbred strains^4^ derived from the LS and SS mice, however, have shown that the α*4 nAChR* polymorphisms are associated with alcohol’s ability to suppress the extent to which mice are startled by a loud noise (i.e., the acoustic startle response) (Owens et al. 2003).

Several other studies have examined the influence of polymorphisms in the α*4 nAChR* gene on other variables related to alcohol and nicotine use. For example, Butt and colleagues (2005) determined that mice expressing the different forms of the α4 nAChR subunit differ in how much they “like” nicotine when given a choice between normal tap water and water containing nicotine. Although the animals also differ in how much they “like” alcohol, this is attributed to a gene nearby the α*4 nAChR* gene and not the result of the gene encoding the α4 nAChR subunit. Lastly, the two forms of the α*4 nAChR* appear to modulate the hyperexcitability that occurs during alcohol withdrawal (i.e., when alcohol is suddenly withheld following long-term administration) (Butt et al. 2004).

#### Polymorphisms in the α7 nAChR Subunits

Researchers also have identified alternate forms of the α*7 nAChR* subunit gene of mice (Stitzel et al. 1996). Unlike the α*4 nAChR* gene polymorphism described above, however, the α*7 nAChR* gene polymorphism does not lead to differences in the α7 nAChR proteins but does lead to differences in the levels of α7 nAChRs that are produced and in the pattern of α*7 nAChR* expression during different phases of development. Because alcohol directly acts on α7 nAChRs and because, as described below, α7 nAChRs appear to be involved in modulating several actions of alcohol, it is possible that these α*7 nAChR* polymorphisms influence the actions of alcohol. However, this hypothesis has not yet been experimentally investigated.

#### Polymorphisms in Other nAChR Subunits

Experiments conducted in mice have indicated that there also may be α*5* and α*6 nAChR* subunit polymorphisms; however, to date it is not clear whether the observed variations are located directly in the α*5* and α*6 nAChR* subunit genes or in the vicinity of these genes. Nevertheless, studies have suggested that these differences modulate differential responses to some of the effects of nicotine (Stitzel et al. 1998; Tritto et al. 2002). Whether they also modulate differential responses to some of the effects of alcohol remains to be determined.

### Studies in Genetically Modified Mice

A powerful molecular biological tool to study the effects of certain genes (e.g., of genes suspected to influence the actions of alcohol and/or nicotine) is the generation of genetically modified mice. Of importance to research into interactions between alcohol and nicotine are primarily two groups of such animals:

- “Knock-out” or null mutant mice, in which a single gene (e.g., the gene for a single nAChR subunit) has been deleted so that these animals no longer produce the protein of the deleted gene. For example, β*2 nAChR* knock-out mice no longer express the β2 nAChR subunit protein and, therefore, do not produce any nAChR subtypes that contain the β2 subunit (e.g., α4β2 nAChRs).
- “Knock-in” mice, in which a single gene is introduced into animals to replace a normal gene. Often, these animals produce a protein that is altered or is produced at higher levels than the original protein.

#### Studies Using “Knock-Out” Mice

Although many different *nAChR* knock-out mice are available, very few studies have used these mice to determine the role of nAChRs in modulating the effects of alcohol or the interactions between alcohol and nicotine. Owens and colleagues (2003) used β*2 nAChR* knock-out mice to determine whether β2-containing nAChRs (primarily α4β2 nAChRs) influence any of the effects of alcohol. This study found that β*2* knock-out mice exhibited reduced sensitivity to alcohol-induced depression of the acoustic startle response described above, suggesting that β2-containing receptors modulate this effect of alcohol.

Wehner and colleagues (2004) studied mice in which the α*7,* β*2,* β*3,* or β*4 nAChR* subunit genes had been knocked out to determine the role of these genes in a specific type of learning that involves an animal’s ability to associate a noxious stimulus with environmental cues. These studies suggested that α7 nAChRs, but not nAChRs containing the other subunits, are involved in modulating alcohol’s ability to disrupt this type of learning.

Also, studies of α*7 nAChR* knock-out mice have demonstrated that this receptor is involved in modulating several behavioral and physiological effects of alcohol (Bowers et al. 2005). Compared with mice that expressed α7 nAChRs, α*7 nAChR* knock-out mice showed the following behaviors in response to alcohol:

- Greater enhancement of activity in a brightly lit circular arena,
- More pronounced hypothermic (i.e., body temperature–lowering) response,
- Longer “sleep-time” (duration of alcohol-induced unconsciousness).

Several other measures of the animals’ response to alcohol did not differ between the knock-out mice and normal animals, indicating that the α*7* gene influences only a subset of alcohol’s effects.

#### Studies Using “Knock-In” Mice

In the study by Owens and colleagues (2003) mentioned above, the researchers also examined mice in which a hyperexcitable α*4 nAChR* subunit gene was “knocked-in.” In these mice, the normal α*4 nAChR* gene was replaced with a gene encoding an α4 nAChR protein that differs at only a single amino acid from normal α*4 nAChR* subunits but produces nAChRs that are many times more sensitive to activation by nicotine than normal α4-containing receptors. As expected, knock-in of this mutant subunit gene had the opposite effect of deleting the β*2 nAChR* gene: Mice expressing the hyperexcitable α*4 nAChR* gene were more sensitive to alcohol’s ability to depress the acoustic startle response than were animals expressing the normal α*4 nAChR* gene.

## Role of nAChRs in Modulating Alcohol-Induced Neurotoxicity

Researchers also have begun to investigate if and how specific nAChR subtypes contribute to and modulate alcohol’s brain-damaging (i.e., neurotoxic) effects. (For more information on the interactions between alcohol and nicotine, as well as between alcohol and nAChRs, in modulating alcohol’s neurotoxic effects, see the article by Funk and colleagues in this issue.) Studies using cultured neurons derived from α*7 nAChR* knock-out mice demonstrated that the absence of α7 nAChR renders neurons more susceptible to alcohol’s toxic effects (de Fiebre and de Fiebre 2005). This observation suggests that α7 nAChRs may somehow protect the cells against the neurotoxic properties of alcohol and complements findings that α7 nAChRs modulate the neurotoxicity associated with alcohol withdrawal (Mulholland et al. 2003).

Other investigators have shown that nicotine can prevent alcohol-induced death of cultured neurons derived from two different brain regions, the cerebellum and cerebral cortex (Tizabi et al. 2003, 2004). These studies also have demonstrated that agents which selectively block either α4β2 or α7 nAChRs can interfere with these protective effects of nicotine. Interestingly, the investigators noted differences in the potency of the blocking agents across brain regions. This suggests that the nAChR subtypes involved in the protective effects of nicotine may differ between the cerebellum and cerebral cortex.

## Conclusion and Future Outlook

Although this review may give the impression that much is known about the nAChRs involved in modulating some of the interactions between alcohol and nicotine, great gaps in knowledge remain regarding these interactions and how they modulate the acute and chronic actions of both drugs. Moreover, researchers still do not know how these interactions modulate the development and maintenance of co-dependence on alcohol and tobacco. Elucidation of these processes is of utmost importance because smoking has been identified as one of the most important risk factors for alcoholism. Despite the importance of smoking/nicotine use in alcoholism, only a small fraction of research into the biology of alcohol has focused on the mechanisms involved in modulating the co-use and abuse of alcohol and nicotine. It is imperative that researchers gain a greater understanding of the role of nAChRs in modulating alcohol–nicotine interactions in order to more clearly understand the factors involved in the development of alcoholism. Such an understanding should lead to more effective treatments for alcohol and/or tobacco dependence.

**Role of Other nAChR Subtypes in Modulating Alcohol’s Effects**

As discussed in the main article, most of the research examining the interactions of alcohol and nicotine in the brain has focused on the two most prevalent subtypes of nAChRs, namely the α4β2 and α7 nAChR subtypes. Recently, however, alcohol researchers also have begun to examine another group of nAChRs that are blocked by a toxin known as α-conotoxin MII (α-CtxMII), which is derived from a type of sea snail. There are two types of these receptors: One type appears to be composed of α4, α6, β2, and β3 nAChR subunits and the other appears to be composed of α6, β2, and β3 nAChR subunits (Salminen et al. 2005). Although relatively little is known about these α-CtxMII nAChRs, they have begun to receive more interest because they appear to be involved, at least in part, in modulating the nicotine- and alcohol-induced release of the neurotransmitter dopamine in a brain region called the nucleus accumbens. Dopamine release in this brain region is thought to be a central event in the brain’s reward system that is stimulated by alcohol, nicotine, and other drugs of abuse.

Over the last decade, researchers in Sweden have investigated the role of nAChRs in modulating the stimulatory and rewarding properties of alcohol (Larsson and Engel 2004). Recently, these investigators examined how α-CtxMII nAChRs modulate the actions of alcohol, specifically the alcohol-induced dopamine release in the nucleus accumbens (Larsson et al. 2004). To this end, the investigators applied α-CtxMII into a brain area called the ventral tegmental area (VTA), where the neurons that release dopamine in the nucleus accumbens originate. The study found that α-CtxMII administration into the VTA reduced alcoholinduced release of dopamine and also reduced the stimulation of locomotor activity by alcohol after α-CtxMII administration to the VTA. Collectively, these data suggest that α-CtxMII nAChRs in the VTA may be involved in modulating the rewarding properties of alcohol.

Although these data are exciting, much remains to be learned about alcohol’s actions at the α-CtxMII nAChRs. For example, researchers do not know yet whether alcohol acts directly at these nAChRs and what the nature of these actions is. Because α-CtxMII nAChRs appear to contain an α6 nAChR subunit, knock-out of the α*6 nAChR* subunit gene would be expected to have pronounced effects on the actions of alcohol. However, such analyses have yet to be conducted. The interaction between nicotine and alcohol at these α-CtxMII nAChRs, as well as the contribution of such interactions to the co-abuse of alcohol and nicotine, also still remain to be examined.

—Tiffany J. Davis and Christopher M. de Fiebre

ReferencesLarssonAEngelJANeurochemical and behavioral studies on ethanol and nicotine interactionsNeuroscience and Biobehavioral Reviews2771372020041501942110.1016/j.neubiorev.2003.11.010LarssonAJerlhagESvenssonLIs an alpha-conotoxin MII-sensitive mechanism involved in the neurochemical, stimulatory, and rewarding effects of ethanol?Alcohol3423925020041590291910.1016/j.alcohol.2004.10.002SalminenOWhiteakerPGradySRThe subunit composition and pharmacology of alpha-Conotoxin MII-binding nicotinic acetylcholine receptors studied by a novel membrane-binding assayNeuropharmacology4869670520051581410410.1016/j.neuropharm.2004.12.011

## Footnotes

1 Plus signs, as in Na^+^ or Ca^2+^, indicate that the ion carries the corresponding number of excess protons compared with electrons; similarly, minus signs, as in Cl^−^, indicate that the ion carries the corresponding number of excess electrons compared with protons.

2 Note that although α1 and β1 subunits of nAChRs exist, they are found in muscle but not in brain (Gotti and Clementi 2004).

3 Desensitization refers to the ability of some drugs to rapidly “turn off” (desensitize) a receptor such that it temporarily can no longer be activated by that drug or other drugs.

4 Recombinant inbred (RI) strains are groups or strains of animals in which the genes of two parental strains (e.g., the LS and SS mice) have been reshuffled (recombined) through the breeding of multiple new strains. Each RI strain has a unique combination of genes from the two parental strains.
